# Eye Movement and Recall of Visual Elements in Eco-friendly Product

**DOI:** 10.16910/jemr.17.4.6

**Published:** 2024-12-06

**Authors:** Jing Li, Myun Kim

**Affiliations:** Department of Marine Design Convergence Engineering, Pukyong National University, Korea

**Keywords:** eye tracking, attention, recall, product design, reading, sustainability graphic, eco-friendly product

## Abstract

This study aims to explore the distribution of visual attention on sustainability graphics when viewing an eco-friendly product and the recall of sustainability information afterward. Twenty-five students majoring in environmental studies and twenty-five students from non-environmental majors participated in the study. They were further divided into a higher group and a lower group based on their sustainability level. Participants viewed diagrams of an eco-trash boat design with sustainability graphics and a 15-page design description. Their eye-movement data and verbal reports on the recall of sustainability information were collected. Higher sustainability group had higher fixation count in sustainability graphics. Non-environmental majors had a shorter time to first fixation to sustainability graphics, and there was an interaction effect. Environmental students detected graphics faster in the lower group, but the opposite occurred in the higher group. Higher-sustainability non-environmental students were quicker, while the reverse was true for environmental students. In terms of recalling sustainability graphics, the higher group scored higher, while environmental majors scored higher in recalling sustainability features. In the recall coding, the most frequently mentioned terms were "green," "plant," "vivid," and "eco." The study offers new insights into sustainable development and provides design recommendations for eco-product designers.

## Introduction

Environmental protection has gained increasing attention worldwide, particularly in the context of sustainable development. As a crucial means of addressing global issues such as climate change and resource depletion, environmental protection not only relies on professionals in relevant fields but also requires enhancing public awareness of sustainability (52). In this process, visual communication, especially visual elements in eco-friendly products, has gradually become a key method for conveying sustainability information (28). In recent years, with the rapid development of eco-friendly product design, researchers have begun to explore how visual elements affect people's understanding and memory of sustainability concepts (20). Eye-tracking technology, as an effective tool for studying visual attention, can reveal how individuals distribute their attention to sustainability graphics when viewing eco-friendly products, providing important insights into the effectiveness of visual communication (44; 49; 51).

Studies have shown that the distribution of visual attention and the recall of information are closely related to an individual's background knowledge and cognitive level. An individual's professional background and cognitive preferences play a critical role in visual information processing (5). In the field of sustainability, this background difference may be even more pronounced, as education in environmental studies can influence an individual's sensitivity to sustainability information and cognitive levels (42). However, there is still a limited amount of research on how individuals with different professional backgrounds and levels of sustainability awareness distribute their visual attention and recall sustainability information when viewing eco-friendly products. Therefore, this study aims to investigate the distribution of visual attention and the recall of sustainability information among individuals with varying professional backgrounds and sustainability levels through eye-tracking experiments.

### Water waste disposal

Water is an essential resource for human survival, playing a crucial role in human health and sustainable development. It is imperative to implement necessary measures to protect water resources and raise awareness about the sustainable development of water resources (22). Waterborne waste has become a significant sustainability issue. Over 2 million tons of plastic are dumped into water bodies each year, ultimately ending up in the oceans (36). Additionally, water contains various types of natural or man-made waste such as food scraps, metals, clothing, and glass. With technological advancements, the methods for managing waterborne waste have evolved beyond manual labor to include specialized products. Low-cost, highly corrosion-resistant, and durable automated waterborne waste management products are now available and effectively perform waste collection and processing tasks (34). The design of these waterborne waste management products can reflect sustainable principles. For example, the design of a waterborne waste management robot uses renewable energy to reduce energy consumption and incorporates sensors and computer vision technology to enhance cleaning efficiency and enable autonomous operation (38). Although these designs are effective, the waste management process and the underlying sustainability aspects are not easily understood by non-specialists. The appearance and design specifications of waterborne waste management products, such as trash boats, often do not intuitively convey sustainability. This lack of intuitive understanding can hinder citizens' comprehension of waterborne waste management practices and the formation of concepts related to the sustainable development of water resources.

### Sustainability graphics in eco-friendly products

With the worsening of global environmental issues and the promotion of sustainable development concepts, the design of eco-friendly products has become a significant trend in contemporary design (6). In eco-friendly products, the design of visual elements not only concerns aesthetics but also carries the mission of conveying sustainability concepts (20). Graphics are an effective way to communicate ideas, methods, and styles (4). Incorporating graphic design into industrial product design can more effectively express the designer's intentions (18). Integrating sustainability elements into graphics and product design is an important approach to promote sustainable development (1). Industrial designers have both the ability and responsibility to raise public awareness of sustainability by not only enhancing the functional sustainability of products but also highlighting sustainability visually to enhance public sustainability education. By using specific visual symbols, colors, and patterns, designers can communicate environmental messages and influence the public's cognition and attitudes. These visual elements help users cultivate environmental awareness in their daily lives, thereby promoting sustainable behaviors (8).

In various fields, sustainability graphics have been proven to play a role in promoting sustainable development. Eco-graphic labels have played an important role in consumers’ food choices (16). The use of sustainability graphics in product packaging not only influences consumers' perceptions and inferences about the product but also affects their sustainability attitudes (45). One study further demonstrated that not only the material type of the packaging affects consumers but also the graphic materials, especially for consumers with lower environmental awareness (16). Additionally, incorporating sustainability elements into electronic graphic interfaces can guide users toward environmentally friendly actions (35). Symbolic visual elements, such as green and plant images, are often associated by consumers with environmental protection and sustainability (37). For example, green labels and eco-symbols not only significantly increase consumers’ awareness of the environmental attributes of products but also enhance consumers' trust and loyalty toward the brand (48).

However, the public's response to these visual elements is not uniform, but rather influenced by their professional background, environmental awareness, and cognitive level. Individuals with an environmental studies background are more likely to identify sustainability visual elements in products and are more receptive to this information compared to the general public (42). Furthermore, individuals' sustainability levels can also influence their consumption behaviors and environmental actions (43). Therefore, studying the differences in how individuals with different professional backgrounds and sustainability levels respond to visual elements in eco-friendly products can help reveal the effectiveness of these visual elements in conveying sustainability concepts.

### Eye-tracking study

Eye-tracking technology is an effective method for exploring graphics and is frequently used in visual and product design (26; 29). Eye-tracking technology can record participants' eye movements, allowing researchers to infer visual attention and attention transaction patterns (33; 46; 53). For example, a study analyzing eye movements on warning labels on alcoholic beverage packaging found that the position, size, and color of the labels significantly influenced consumer visual attention (9)). Fixation is a common eye movement in eye-tracking research, referring to the behavior of the foveal vision staying in a specific area for more than a certain amount of time (23). Although the human field of view is broad, only the positions of fixations are the actual targets of individual attention(19). Eye-tracking metrics related to fixation include fixation duration, fixation count, and time to first fixation. Fixation duration and fixation count are generally interpreted as measures of attention, while time to first fixation reflects the ability of a target to quickly attract attention (21). In eye-tracking research, an area that contains key experimental objects can be defined on the material, and this area is referred to as the Area of Interest (AOI). Its primary function is to mark the areas that need to be analyzed in the study, allowing for the analysis of eye-tracking metrics within single AOI and across multiple AOIs (2; 25; 32).

In sustainability research, eye-tracking technology has also been extensively used. One study focused on consumers' eye movements regarding in-store recycling signage, collecting data on fixation duration, fixation count, and time to first fixation(55). The results indicate that the total fixation duration (1.72 s) and fixation count (5.91 counts) for shelf strips performed better than those for aisle invaders and store drop-off bin panels. However, for time to first fixation (0.12 s), store drop-off bin panels showed the best performance. This indicates that there are differences in the appeal of sustainability visual elements to users. In a study on meat sustainability, researchers analyzed consumers' eye movements and found that the Free-Range logo received the highest attention, as reflected in the average fixation count (4.03 counts), average fixation duration (0.89 s), and average visit duration (0.95 s) (14). Through further interviews, researchers found that consumers often make incorrect inferences about sustainability labels, frequently associating higher prices with greater sustainability. This highlights the crucial role of graphics in conveying sustainability concepts, as well as the shortcomings in the design of graphics within related products. In the field of sustainable heritage, eye-tracking demonstrated that the diverse cultural backgrounds of monuments did not lead to significant differences in visual responses among observers from different ethnicities (41). A study utilizing eye-tracking technology in the sustainability education of industrial design students revealed that students employed different strategies and performances when reading sustainability articles (31).Specifically, students with a higher level of sustainability scored higher on perspective scores, but there was no difference in recall scores. They also had a longer fixation duration on images compared to the lower group. Furthermore, the fixation transition patterns differed between theoretical articles and case articles; in case articles, students showed more transitions between fixations on images. Eye-tracking technology has been validated for its reliability in sustainability research and has shown great potential in understanding visual cognitive behaviors and patterns, addressing the limitations of subjective data in this field (28; 45; 55). These studies provide new insights into sustainability research.

### The present study

Although many studies have explored the importance of visual elements in product design and their influence on user behavior (20; 54), research on the impact of sustainability visual elements in eco-friendly products on individuals with different professional backgrounds and levels of sustainability awareness remains limited. Most eye-tracking studies in the sustainability domain have focused on areas such as food packaging and electronic graphic interfaces, with relatively little attention given to how sustainability visual elements in eco-friendly products affect consumers' visual attention distribution and information recall. Moreover, while some studies have examined the relationship between consumers' environmental awareness and their attitudes toward eco-friendly products (7; 43), there is still a lack of systematic research into how individuals from environmental and non-environmental backgrounds differ in their visual responses to sustainability visual elements in eco-friendly products. These differences not only affect the distribution of visual attention but may also influence consumers' memory and information processing.

This study aims to fill the aforementioned research gap by exploring the differences in visual attention distribution and information recall among individuals with different professional backgrounds and sustainability levels when confronted with sustainability visual elements in eco-friendly products. Given the lack of research on the application of sustainability graphics in waterborne waste management products, we designed an eco-trash boat and applied sustainability graphics to both the boat design and the accompanying design description. Through the use of eye-tracking technology, this study will provide empirical data to reveal the differences in attention focus and information processing between environmental and non-environmental students regarding sustainability visual elements.

This study will help address the current research gap on the role of visual elements in eco-friendly product design, offering empirical evidence for future research. The results of this study will provide crucial design recommendations for eco-friendly product designers, helping them understand how to optimize visual elements based on the professional backgrounds and sustainability awareness levels of different user groups. By creating more appealing and memorable sustainability graphics, designers can more effectively communicate environmental messages. In the context of global sustainable development, the promotion of eco-friendly products has become an important task. This study, by revealing the differences in users' cognition and recall of sustainability visual elements, will provide valuable insights for raising public environmental awareness and promoting green consumer behavior. This, in turn, will help foster broader environmental consciousness and contribute to the achievement of sustainable development goals.

This study proposes three research questions (RQs):

RQ1: Do major and sustainability level have main effects and interaction effects on eye movement (fixation count and time to first fixation)? We expect differences in eye movement characteristics depending on professional background and sustainability level. Specifically, we hypothesized that environmental students and students with higher sustainability levels will have a higher fixation count on sustainability graphics and will detect sustainability graphics more quickly.

RQ2: Do major and sustainability level have main effects and interaction effects on the recall of sustainability elements (sustainability graphic and sustainability feature)? We expect differences in the recall of sustainability elements depending on professional background and sustainability level. Specifically, we hypothesized that students with higher sustainability levels will have an advantage in recalling sustainability graphics, while environmental students will have an advantage in recalling sustainability features.

RQ3: What aspects did participants mention when recalling the sustainability graphics? We hypothesized that participants will more frequently mention nature-related and design-related attributes of the sustainability graphics.

## Methods

### Participants

The sample size was calculated using G*Power 3.1.9.7. The test family was set to F tests, the statistical test to ANOVA: Fixed effects, special, main effects and interactions, with an effect size f of 0.45, an α error probability of 0.05, and a power of 0.8. The numerator degrees of freedom were set to 1, and the number of groups to 2. This resulted in a total sample size of 41. Since 41 cannot be evenly divided into groups, the minimum number of participants required was therefore set to 42. This study recruited a total of 50 volunteers (20.5±2.33 years old) to participate in the experiment, including 29 males and 21 females. They were all undergraduate students, with 25 participants majoring in environmental studies and the other 25 majoring in non-environmental studies. The participants had not seen the materials used in the experiment before participating. All had normal or corrected-to-normal vision. Before data collection, all participants were informed that they would be participating in a study involving reading a sustainable product design proposal and design description, and that their eye movements and verbal reports would be recorded. All participants signed an informed consent form before the experiment. Each participant received a gift worth 5 dollars as compensation.

### Stimuli

**Figure 1.**
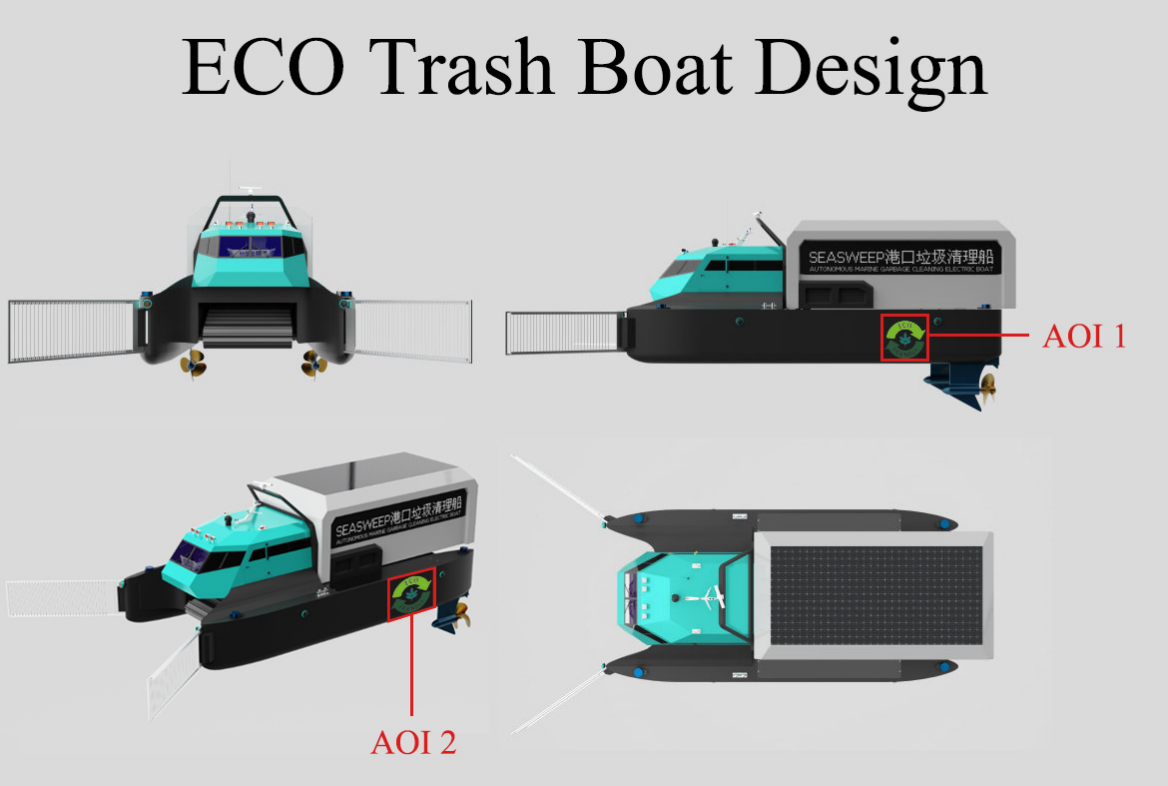
Four views of the eco-trash boat design (the red lines and AOI instructions were not visible to participants)

**Figure 2.**
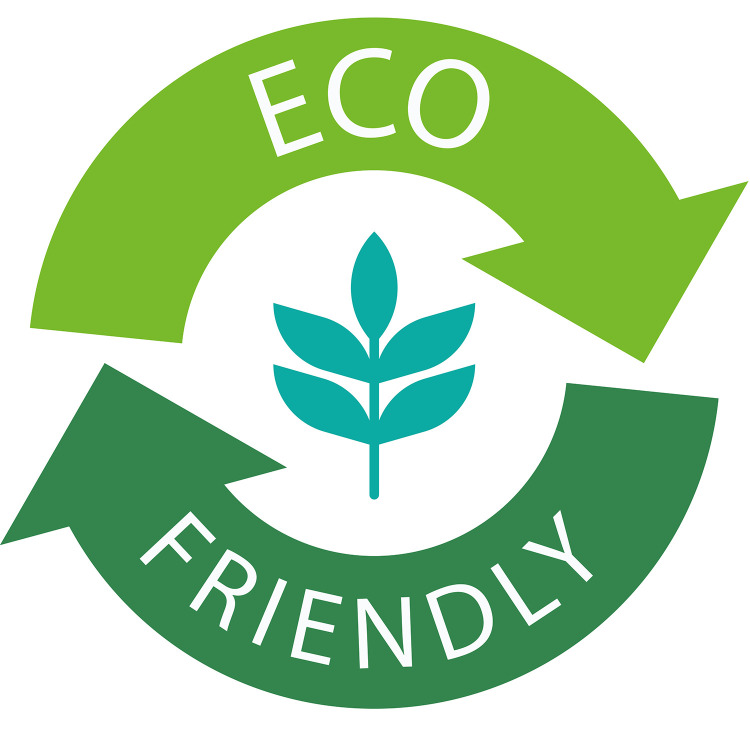
Sustainability graphic in the eco-trash boat design

The experiment included two stimuli. Stimulus 1 was a page displaying four views of the eco-trash boat design (Figure 1), presented on a monitor. The eco-trash boat was designed by the researchers to handle waterborne waste and enhance environmental sustainability. On the side of the eco-trash boat, there was a sustainability graphic, which is designed by the researchers (Figure 2), visible in both the right and perspective views. The areas containing the sustainability graphic in the four-view page were designated as AOI 1 and AOI 2 (Figure 1). These AOIs were defined during data processing for analyzing eye movements, but participants were neither shown nor informed about the AOI regions. Stimulus 2 was a 15-page design description, printed in color. The design description of the eco-trash boat aimed to provide participants with a comprehensive understanding of the product's appearance, functionality, principles, and sustainability concepts. One page is the title, one page displays the dimensions, one page shows the overall render, one page presents the structure map, seven pages are dedicated to function introduction, and four pages illustrate usage scenarios (Appendix).

### Instruments

Participants' self-assessed sustainability levels were measured using a subjective scale (17). This scale is primarily used to measure participants' levels of sustainable consumption and is divided into eight dimensions: activism, personal sacrifice, communitarianism, environmental concern, healthy food, perceived consumer effectiveness, search for information, and social concern. The scale consists of 31 questions, using a 7-point Likert scale where 1 represents "strongly disagree" and 7 represents "strongly agree." The total score is standardized and converted to a final score out of 100. This scale has been validated in studies on sustainable design among undergraduate students (31). In this study, a pilot test of the scale resulted in a Cronbach's alpha value of 0.89.

Participants' eye movement data were recorded using Tobii Pro Glasses 2. The monitor presenting the four-view design was 21 inches (resolution of 1920×1200). The design description was printed on A4 paper. Participants' verbal reports were recorded using an HP-XXJ1 and transcribed into txt format by the researchers.

### Procedure

**Figure 3.**
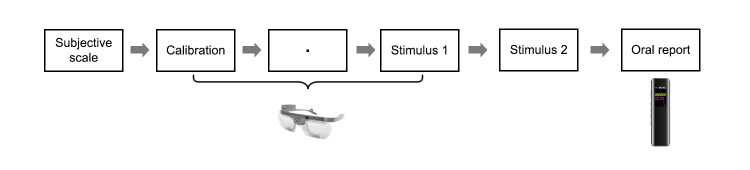
Experimental process

Participants were informed about the purpose of the study and the experimental procedure, and after signing the informed consent form, they recorded their gender, age, and major. The entire experiment took approximately 30 to 45 minutes (Figure 3). Participants first completed the sustainable consumption measurement scale. Once ready, participants wore the Tobii Pro Glasses 2 and underwent calibration. After that, they sat in front of the screen to prepare for viewing the experimental stimuli. The screen displayed instructions for the experimental procedure, and participants clicked the space bar when ready. A black dot appeared in the center of the screen for 1000ms, followed by the four-view design of the eco-trash boat. After thoroughly viewing the design, participants clicked the space bar again, and the screen displayed "Thank you for watching, please remove the eye-tracker," signaling the end of eye-tracking data collection. The average viewing time of the eco-friendly boat design is 387.1 seconds (SD=110.21). Participants then removed the eye-tracker. Next, the researcher provided the participants with the design description of the eco-trash boat. Participants sat at a table and had up to 10 minutes to read the design description. After reading, participants had up to 5 minutes to verbally report the sustainability elements they found in the four-view design and the design description, which the researcher recorded using an HP-XXJ1.

### Data analysis

The independent variables include major (environmental, non-environmental) and level of sustainability (higher, lower). Based on the sustainability scores, the top 25 participants were defined as the higher group, and the bottom 25 participants were defined as the lower group. The dependent variables include fixation count, time to first fixation, sustainability graphic, and sustainability feature. The eye-movement data were processed and exported using Tobii Pro Lab (Tobii Pro, Stockholm, Sweden). The criteria for identifying fixations were set as those lasting more than 200 milliseconds and within a diameter of no more than two degrees. Eye-tracking data samples with a collection rate below 90% were excluded. The total number of fixations on AOI 1 and AOI 2 combined represents the fixation count on the sustainability graphic. The smaller of the first fixation times for AOI 1 and AOI 2 is taken as the time to first fixation for the sustainability graphic. Scores for sustainability graphic and sustainability feature are obtained through coding and scoring. In the verbal reports, correct mentions or discussions of visual elements related to sustainability in the eco-trash boat design were coded as visual elements, with each occurrence adding one point to the sustainability graphic score. Correct mentions or discussions of functions or mechanisms related to sustainability were coded as functional elements, with each occurrence adding one point to the sustainability feature score. Table 1 provides an example of coding and calculating scores for sustainability graphic and sustainability feature. For instance, if a participant mentioned sustainability-related visual elements three times and sustainability-related functions three times in their verbal report, both the sustainability graphic and sustainability feature scores would be 3. Two researchers independently coded the text of the participants' verbal reports. In case of discrepancies, discussions were held to resolve them. If disagreements persisted, a third expert made the final judgment.

**Table 1.** An example of coding and scoring sustainability graphics and sustainability features.

Variable	Oral report	Score
Sustainability graphic	"The trash disposal boat has a green icon that immediately indicates it is related to environmental protection." (sustainability graphic#1)	3
	"The graphic for the circular economy is very intuitive and easy to understand." (sustainability graphic#2)	
	"The green leaf feels very natural." (sustainability graphic#3)	
Sustainability feature	"Solar panels can effectively utilize renewable resources, thereby saving energy." (sustainability feature#1)	3
	"Biodegradable waste can be converted into plant fertilizer or animal feed, which I think is a very good design." (sustainability feature#2)	
	"This boat can achieve zero emissions, which is very beneficial for sustainable development." (sustainability feature#3)	

Quantitative data were analyzed using SPSS for Windows v. 26.0 (IBM, Armonk, NY, United States). Descriptive statistics for sustainability scores, fixation count, time to first fixation, sustainability graphic, and sustainability feature were reported using means (M) and standard deviations (SD). Differences in sustainability levels between the high and low groups were tested using independent samples t-tests. This is a 2x2 between-subjects design; therefore, a Two-Way ANOVA was used to analyze the main effects and interactions with major and sustainability level as independent variables, and fixation count, time to first fixation, sustainability graphic, and sustainability feature as dependent variables. The LSD method was selected for post hoc testing. Significance was set at p < .05.

## Results

The mean sustainability score for participants was 69.0 (SD = 14.62). Based on the sustainability scores, the top 25 participants, classified as the higher group, had an average score of 81.2 (SD = 5.21), with 16 environmental majors and 9 non-environmental majors. The bottom 25 participants, classified as the lower group, had an average score of 56.8 (SD = 9.93), with 9 environmental majors and 16 non-environmental majors. There was a significant difference in sustainability scores between the higher and lower groups, *t* (48) = -10.895, *p* < .001.

To address RQ1, eye movement metrics, including fixation count and time to first fixation, were analyzed for participants with different majors and sustainability levels (Table 2).

**Table 2.** Descriptive statistics of fixation count and time to first fixation for participants with different majors and sustainability levels.

Group		Fixation count		Time to first fixation (ms)	
		M	SD	M	SD
Non-environmental	Lower group	2.9	1.36	2489.1	271.21
	Higher group	4.7	1.32	1467.9	234.31
Environmental	Lower group	2.4	1.23	1898.3	241.3
	Higher group	4.6	1.31	3495.1	325.17
Total		3.7	1.61	2520.9	811.45

**Figure 4.**
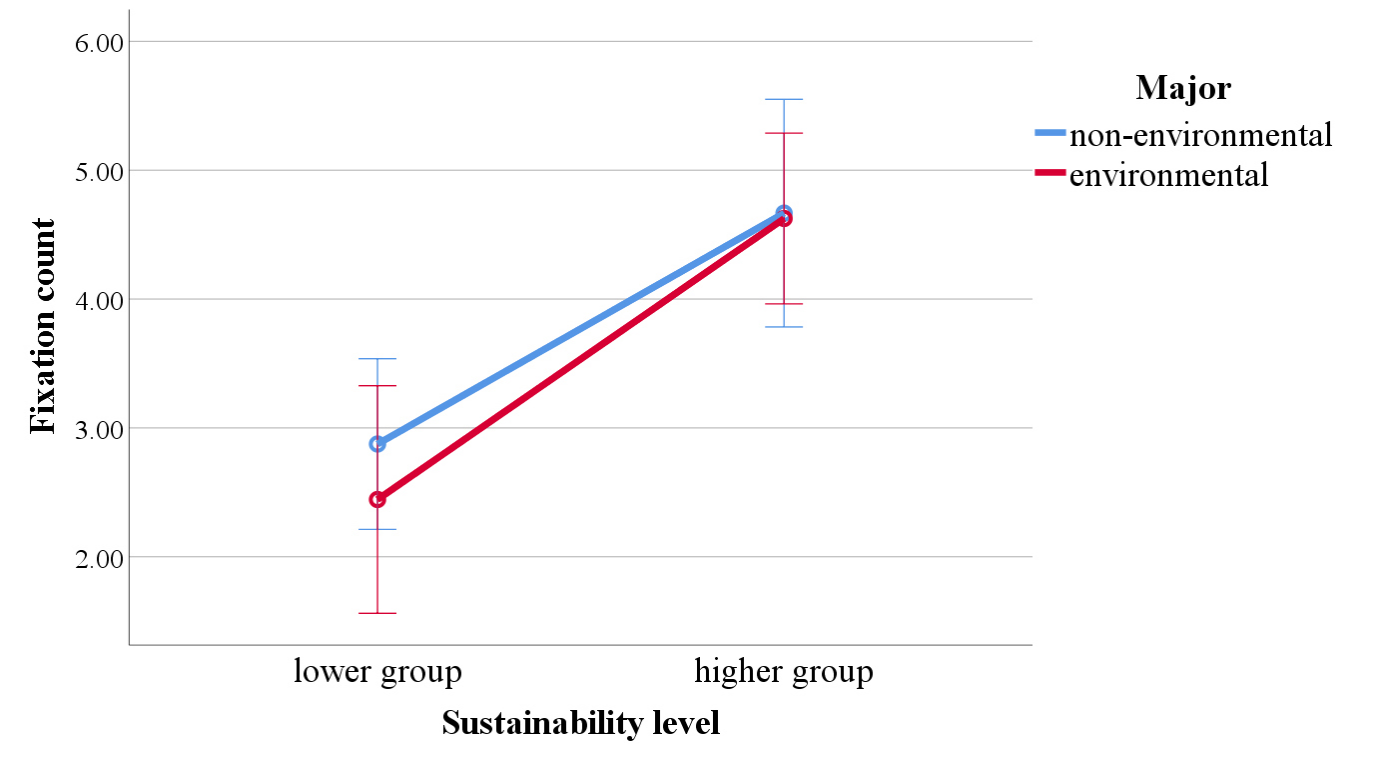
Differences in fixation count depending on major and sustainability level.

In the fixation count results, the main effect of major was not significant, *F* (1, 46) = 0.371, *p* = .546, *η²* = .008. The main effect of sustainability level was significant, with the fixation count in the higher group being significantly greater than that in the lower group, *F* (1, 46) = 26.220, *p* < .001, *η²* = .363. The interaction effect between major and sustainability level was not significant, *F* (1, 46) = .251, *p* = .619, *η²* = .005 (Figure 4).

**Figure 5.**
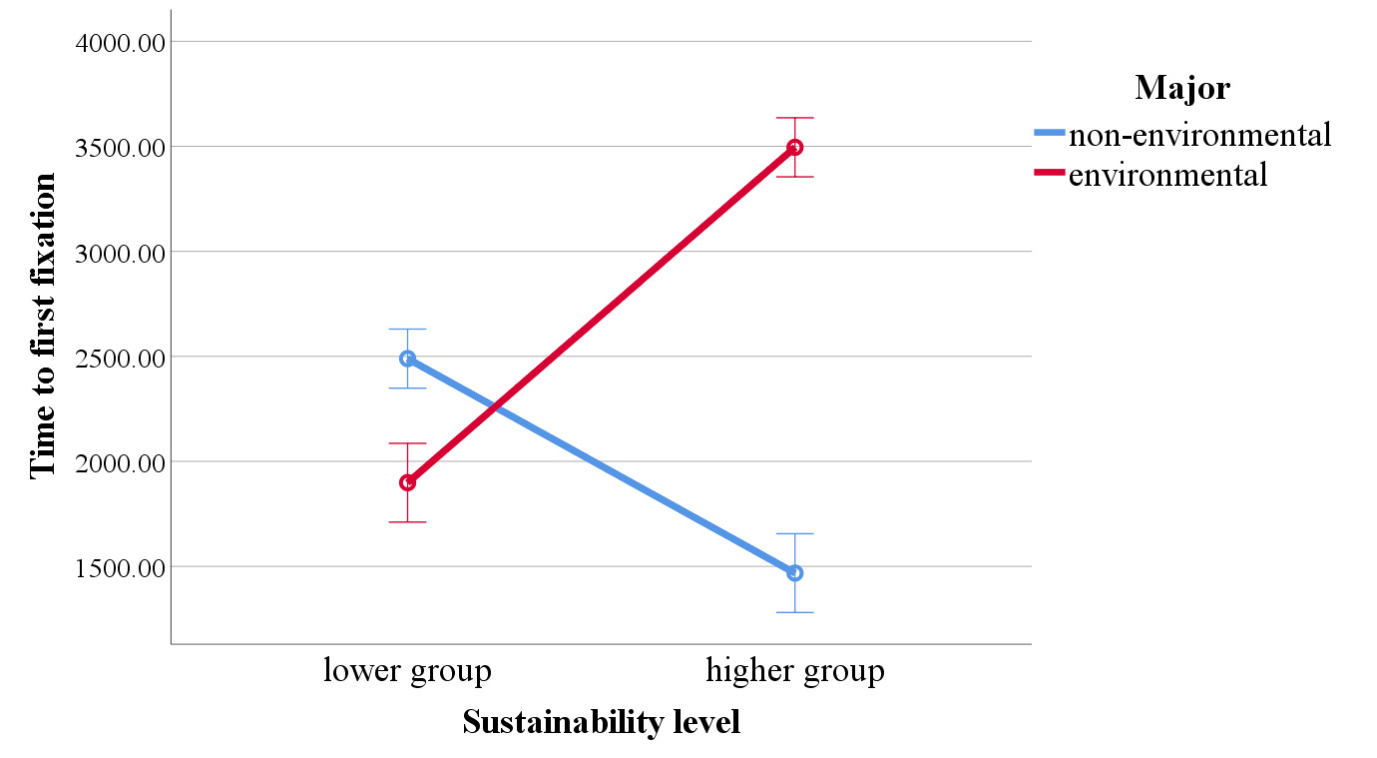
Differences in time to first fixation depending on major and sustainability level.

In the results for time to first fixation, the main effect of major was significant, with non-environmental participants having a significantly shorter time to first fixation than environmental participants, *F* (1, 46) = 76.051, *p* < .001, *η²* = .623. The main effect of sustainability level was also significant, with the lower group having a significantly shorter time to first fixation than the higher group, *F* (1, 46) = 12.210, *p* < .001, *η²* = .210. The interaction effect between major and sustainability level was significant, *F* (1, 46) = 252.624, *p* < .001, *η²* = 0.846 (Figure 5).

Simple effects results show that in the lower group, non-environmental participants had a significantly longer time to first fixation than environmental participants, *F* (1, 46) = 25.729, *p* < .001, *η²* = 0.359. In the higher group, non-environmental participants had a significantly shorter time to first fixation compared to environmental participants, *F* (1, 46) = 302.945, *p* < .001, *η²* = .2868. Among non-environmental students, the lower group had a significantly longer time to first fixation than the higher group, *F* (1, 46) = 76.879, *p* < .001, *η²* = .626. Conversely, among environmental students, the lower group had a significantly shorter time to first fixation than the higher group, *F* (1, 46) = 187.9549, *p* < .001, *η²* = .803.

To address RQ2, the scores for recall of sustainability elements, including sustainability graphic and sustainability feature, were analyzed for participants with different majors and sustainability levels (Table 3).

**Table 3.** Descriptive statistics of sustainability graphic and sustainability feature by major and sustainability level for participants with different majors and sustainability levels.

Group		Sustainability graphic		Sustainability feature	
		M	SD	M	SD
Non-environmental	Lower group	1.1	0.81	2.1	0.68
	Higher group	3.3	0.87	1.8	0.67
Environmental	Lower group	0.9	0.78	4.0	0.71
	Higher group	2.7	1.08	3.7	1.08
Total		2.0	1.04	2.9	1.24

**Figure 6.**
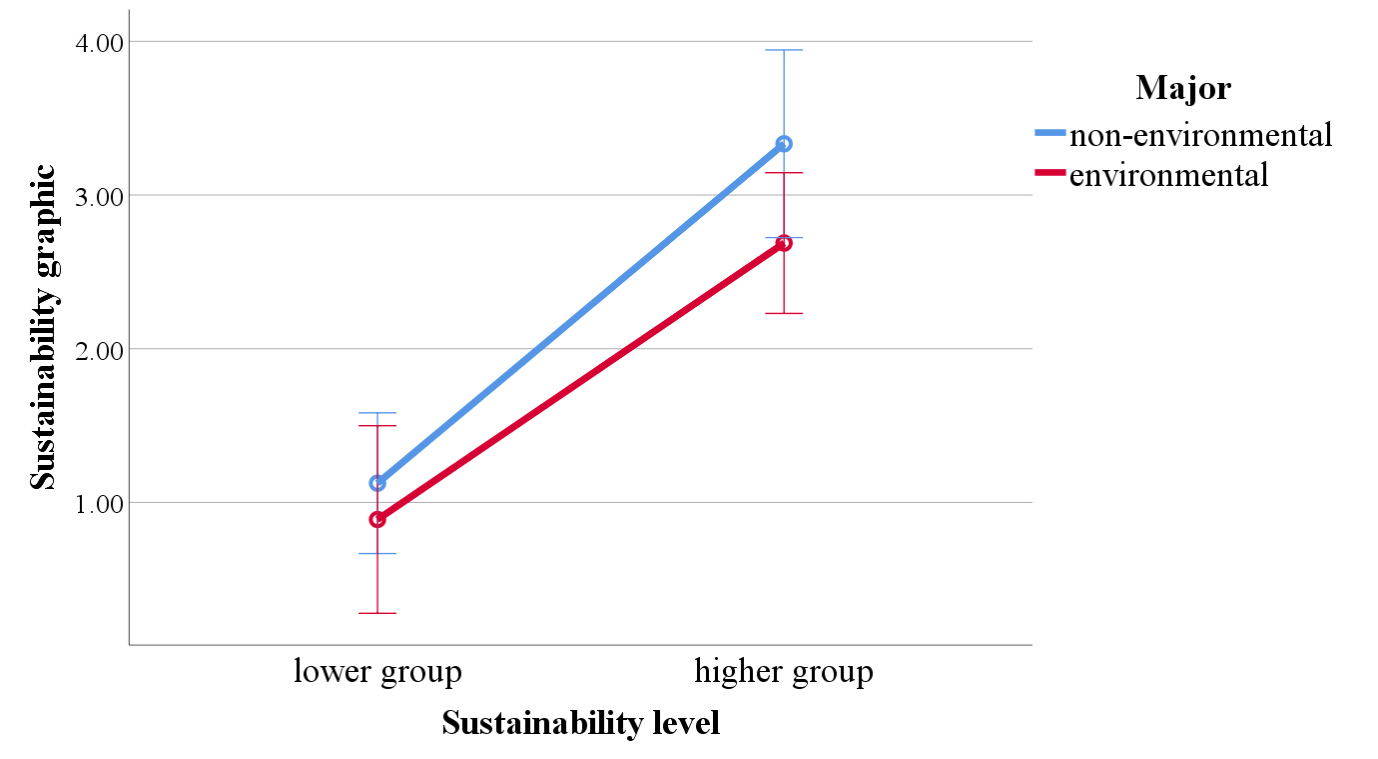
Differences in sustainability graphic depending on major and sustainability level.

In the results for sustainability graphic, the main effect of major was not significant, *F* (1,46) = 2.706, *p* = .107, *η²* = .056. The main effect of sustainability level was significant, with the higher group recalling significantly more sustainability graphics than the lower group, *F* (1,46) = 55.863, *p* ＜ .001, *η²* = .548. The interaction effect between major and sustainability level was not significant, *F* (1,46) = .584, *p* = .449, *η²* = .013 (Figure 6).

**Figure 7.**
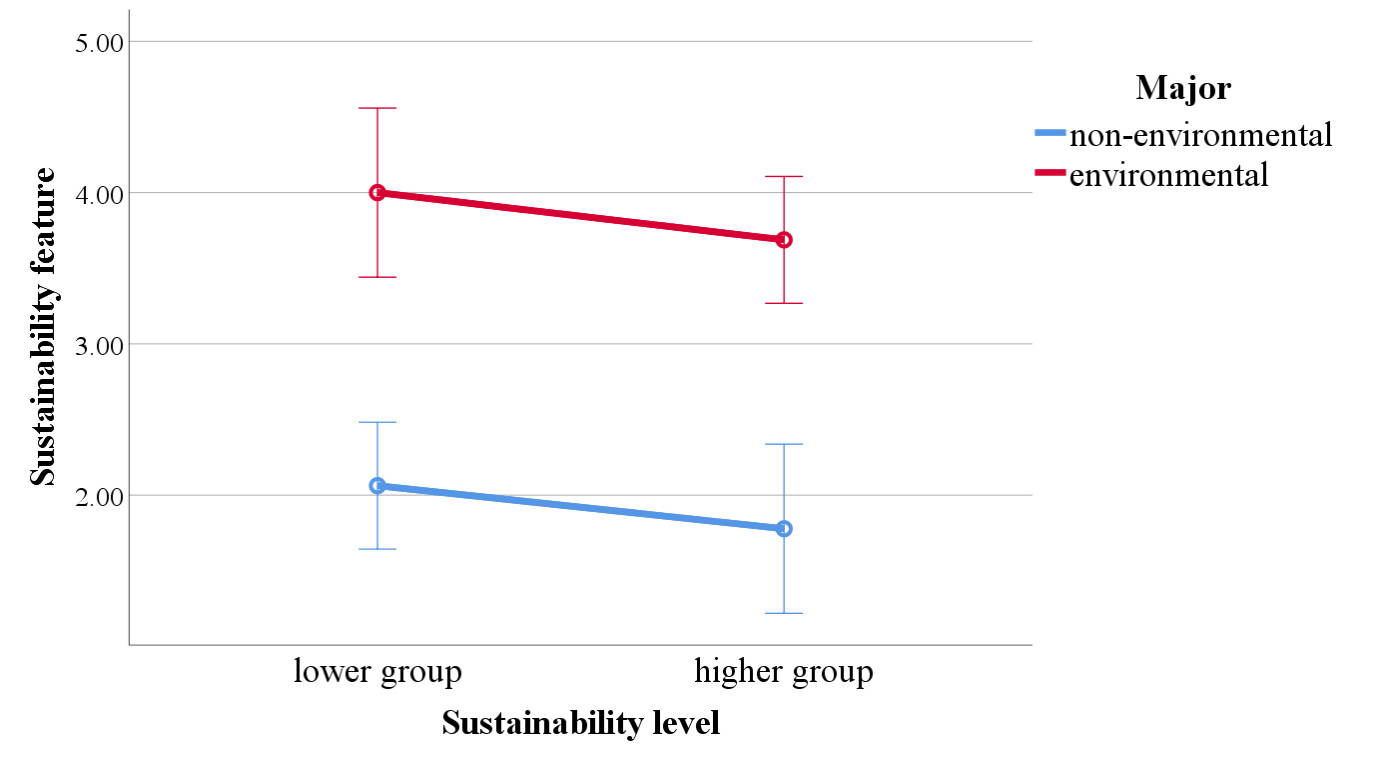
Differences in sustainability feature depending on major and sustainability level.

In the results for sustainability feature, the main effect of major was significant, with non-environmental participants recalling significantly fewer sustainability features compared to environmental participants, *F* (1,46) = 61.410, *p* ＜ .001, *η²* = .572. The main effect of sustainability level was not significant, *F* (1,46) = 1.480, *p* = .230, *η²* = .031. The interaction effect between major and sustainability level was not significant, *F* (1,46) = .003, *p* = .955, *η²* ＜ 0.001 (Figure 7).

**Figure 8.**
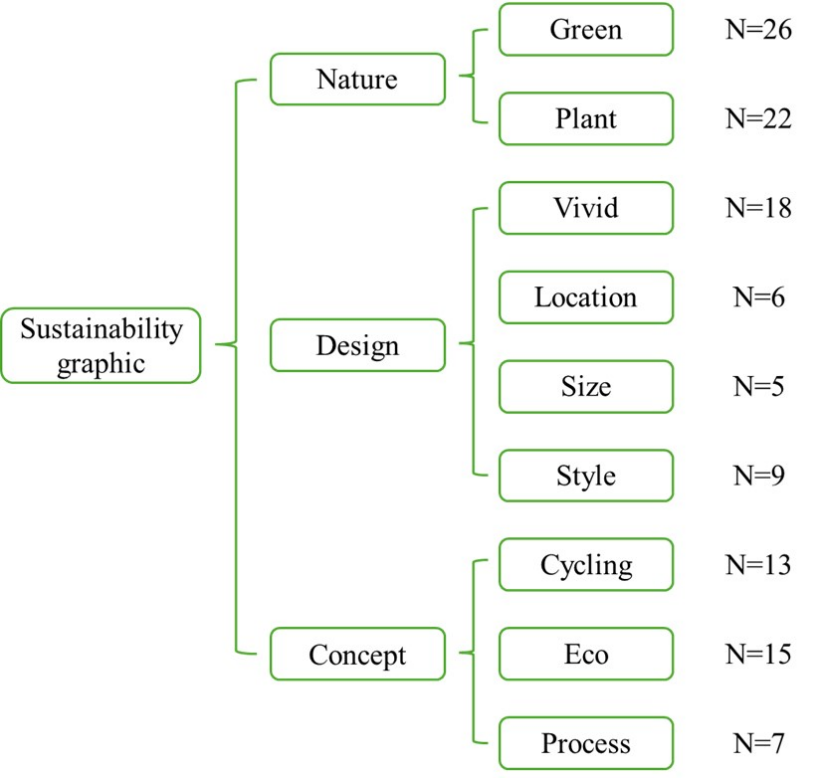
Coding and classification of sustainability graphic (N represents the number of participants who mentioned this sub-category).

To address RQ3, the coding of sustainability graphics was organized and categorized into 3 main categories: nature, design, and concept, and further divided into 9 sub-categories (Figure 8). Nature was mentioned by all participants. The most frequently mentioned sub-category was green, followed by plant, vivid, and eco. For example, one participant mentioned, “I saw a green symbol with two arrows indicating recycling, and a green leaf in the center.” This statement referenced green, plant**, and cycling. Another participant said, “The green image on the boat was particularly striking. It was very vividly designed, allowing people to intuitively feel that the boat is something related to environmental protection.” Here, the coding includes green, vivid, and eco. Some participants specifically commented on the design of the graphic, such as, “There’s an icon on the lower side of the boat showing a green leaf with recycling arrows. However, the graphic’s size is a bit small, which might make it hard for people to see from a distance.” This statement corresponds to green, plant, cycling, and also location and size in terms of design. Style and process were also mentioned, for instance, “The icon is very simple, like elements seen on the packaging of some eco-friendly products,” and “These two arrows make me understand that the waste on the water can be processed into something useful.”

## Discussion

This study aims to explore the eye movements of students with different majors and sustainability levels when viewing sustainability graphics in eco-friendly products, as well as their recall of sustainability elements. This study begins with the product design of water-based sustainability products, focusing on functional and visual optimization of the eco-trash boat, and further discusses the design’s impact on public awareness of sustainability. The study employed a mixed-method approach, collecting both quantitative eye-movement data and qualitative analysis of participants' verbal reports. In the sustainability grouping of participants, the higher group had a larger proportion of students majoring in environmental fields, which was expected. Environmental students receive more education related to sustainability and tend to have a higher awareness of it (10; 15). However, being an environmental major cannot be the sole criterion for determining sustainability levels, and the study results indicate that major and sustainability level have different effects on some measures and may interact. Overall, sustainability graphics have an impact on both visual attention and recall. The specific discussions are provided below.

The eye-movement data was used to address RQ1. We hypothesized that environmental students and higher sustainability students would have a higher fixation count and a shorter time to first fixation on sustainability graphics. The degree of attention to the sustainability graphic in the design's four views can be reflected by fixation count ((30; 47; 55). Students with higher sustainability levels showed more visual attention to the sustainability graphic. However, there was no significant difference between environmental and non-environmental majors. This result indicates that visual elements related to sustainability are more likely to attract the attention of the higher group. This is because the higher group has a greater sensitivity to sustainability, which is reflected in their increased fixation count (27). Sustainability graphics effectively convey awareness of environmental protection, energy conservation, and emission reduction (16; 40). For individuals with already high sustainability levels, this communication is even more effective as they pay more attention to the sustainability graphics. Consumers who spend more time focusing on sustainability elements are more likely to value sustainability and engage in sustainable practices (50). This result further demonstrates that the appeal of sustainability graphics is independent of whether a person is an environmental major. Therefore, the application of sustainability graphics on water-based sustainability products should be designed for the public, not just for those in environmental fields.

An important role of sustainability graphics should be their ability to help people quickly notice sustainability information. Generally, a shorter time to first fixation indicates that participants detected relevant information more quickly (13; 24; 55). The results for time to first fixation are more complex than for fixation count, with both major and sustainability level influencing time to first fixation, and an interaction effect observed. Non-environmental students detected the sustainability graphic earlier than environmental students, and students with lower sustainability levels detected the sustainability graphic earlier than the higher group. This result is somewhat surprising and contradicts our pre-experiment hypothesis. Further analysis of the interaction effect revealed that, in the lower group and among non-environmental students, the results align with our hypothesis, which is higher sustainability levels and being an environmental major facilitate earlier detection of the sustainability graphic. However, in the higher group and among environmental students, the results were contrary. We speculate that this interaction effect is related to participants' knowledge base and the specialized information presented in the eco-trash boat design. The eco-trash boat’s design includes not only sustainability graphics but also aspects like materials, working principles, and operational methods, which may be unfamiliar to non-environmental students or those with lower sustainability levels. Environmental students and higher group participants, due to their higher sustainability literacy (15), might focus on more specialized sustainability information, such as materials and functionality (16), and therefore might not detect the sustainability graphic as quickly. Combining the results for fixation count, despite lower group students not having a higher number of observations, the sustainability graphic was able to capture their attention more quickly. Effective communication of sustainability is crucial, particularly in contexts with a large amount of visual information or insufficient observation time.

To address RQ 2, participants' recall of sustainability graphic and sustainability feature information was encoded and analyzed. Before the experiment, we hypothesized that higher sustainability students would achieve higher recall scores for sustainability graphics, while environmental students would score higher in recalling sustainability features. The results indicate that the higher group reported more information related to the sustainability graphic compared to students with lower sustainability levels, with no significant differences between majors. Environmental majors, however, demonstrated an advantage in the sustainability feature scores. For environmental students, their education provides them with a better knowledge base related to sustainability (10; 15). Thus, when reading the design description, they can easily understand sustainability principles, functions, and implementation methods, leading to higher scores in sustainability features. This professional advantage is not influenced by the level of sustainability awareness. In contrast, the results reaffirm that being an environmental major is not a key factor for sustainability graphic recall. Sustainability awareness not only affects attention to sustainability elements but also impacts understanding and recall of these elements (28). Individuals with higher sustainability awareness are more proactive in seeking sustainability-related information (31), allowing them to recall more content related to sustainability graphics. This result aligns with the fixation count findings, further supporting that the higher group allocated more attention to sustainability graphic-related information and demonstrated better understanding and recall of it.

The categorization results of the coding for sustainability graphic-related information in all participants' oral reports address RQ 3. We aim to interpret which aspects participants focused on by coding their recall of sustainability graphics. The use of green plant imagery in sustainability graphics made a strong impression on participants and was the most frequently mentioned. Visual elements related to nature are helpful for expressing and communicating sustainability (11), as demonstrated again in this study. In the design of sustainability graphics, participants more frequently mentioned vividness rather than specific design specifications. This reflects the role of visualization in enhancing impressions in visual communication design (12). Vivid visual elements are crucial in sustainability visual expression (3; 39). They enable viewers to make more associations with environmental friendliness, aiding in internalizing sustainability concepts. The purpose of using sustainability graphics in products is to convey sustainability ideas or knowledge, making the reception of concepts an important effect. In this study, concepts like cycling and eco were mentioned by nearly half of the participants in relation to sustainability graphics, supporting the effectiveness of graphics in information transmission (54). The concepts conveyed through sustainability graphics contribute to sustainable development (50). For individuals, especially those without an environmental background, there are few opportunities for formal sustainability education. Therefore, incorporating sustainability graphics into products is a significant educational approach. This type of education has low learning costs and can be seamlessly integrated into daily life.

In summary, sustainability graphics on the eco-friendly product can capture people's attention and influence their reception of sustainability information. This impact is significant for both higher and lower sustainability individuals. People with higher sustainability levels tend to engage with sustainability graphics more frequently, while those with lower sustainability levels are attracted to these graphics more quickly. The effectiveness of sustainability graphics is independent of the participant's major, indicating that these graphics are intended for the general public, not just those in environmental fields. Although non-environmental majors may lack specialized knowledge in sustainability, the concepts embedded in sustainability graphics can be effectively conveyed through visual observation. Furthermore, cultivating students' sustainability awareness is crucial (31), and educators should pay attention to this aspect. In designing sustainability graphics, it is recommended to use natural elements and adopt more concrete design approaches. Sustainability graphics are essential for conveying concepts and serve as a crucial method for designers to communicate sustainability information, making it an important focus in design.

This study has certain limitations. Firstly, the stimuli used in the research, the eco-trash boat designed by the researchers, may not represent all types of water-based sustainable products, and the sample size is limited (although the set sample size was achieved, the values for effect size, error probability, and power were relatively lenient.), which restricts the generalizability of the findings. Secondly, while sustainability graphics were used in this study, there was no control group for comparative analysis. Third, regarding eye-tracking data, we only collected data on participants' eye movements while viewing the diagrams, but not while reading the design description. This additional, complex data could have been helpful in interpreting the participants' recall results. Lastly, the qualitative data was solely derived from participants' verbal reports, lacking deeper exploration. In future research, we plan to validate our findings across a broader range of eco-friendly products and apply sustainability graphics in various ways, including adding control groups to further explore the effects of sustainability graphics. In the collection and analysis of eye-tracking data, adopt more comprehensive and appropriate metrics, and include eye-tracking data from participants' reading processes. Additionally, we will conduct targeted interviews with participants to uncover more effective information, investigate the reasons behind eye-movement behavior and sustainability awareness, and gather related suggestions and expectations. We will also place greater emphasis on the correlation between eye-tracking data and qualitative data, which will help in better understanding user behavior.

### Conclusion

This study demonstrates that using sustainability graphics on the eco-friendly product is both effective and necessary. The visual appeal and effectiveness of sustainability graphics in conveying sustainability information are not influenced by participants' field of study. Eye-movement data indicate that even non-environmental professionals are attracted to sustainability graphics. Students with higher sustainability awareness tend to focus more on sustainability graphics, while those with lower sustainability awareness are quicker to notice them. The speed at which sustainability graphics are noticed is also affected by the interaction between field of study and sustainability level. In terms of recalling sustainability elements, participants in the higher group also showed an advantage in recalling information related to sustainability graphics. Attributes related to nature were the most easily recalled, and the vivid imagery of sustainability graphics was frequently mentioned, providing valuable design insights. The study further validates the effectiveness of sustainability graphics in conveying sustainability concepts. Thus, incorporating sustainability graphics in eco-friendly products will positively attract public attention and effectively communicate sustainability concepts, contributing to sustainable development.

### Ethics and Conflict of Interest

The author(s) declare(s) that the contents of the article are in agreement with the ethics described in http://biblio.unibe.ch/portale/elibrary/BOP/jemr/ethics.html and that there is no conflict of interest regarding the publication of this paper. This study was conducted in accordance with the guidelines of the Declaration of Helsinki. The experiment would not cause any mental injury to the participants, have any negative social impact, or affect the participants’ subsequent behaviors. According to the institutional guidelines of Pukyong National University, there was no need to submit material for ethical review.

### Acknowledgements

This work was supported by a grant from Brain Korea 21 Program for Leading Universities and Students (BK21 FOUR) MADEC Marine Designeering Education Research Group.

## Appendix


