# Supplementary material for: Eye Movement and Recall of Visual Elements in Eco-friendly Product
Source: J Eye Mov Res. 2024 Dec 6;17(4):10.16910/jemr.17.4.6. doi: 10.16910/jemr.17.4.6 (PMC11734355; doi:10.16910/jemr.17.4.6)

ECO Trash Boat Design

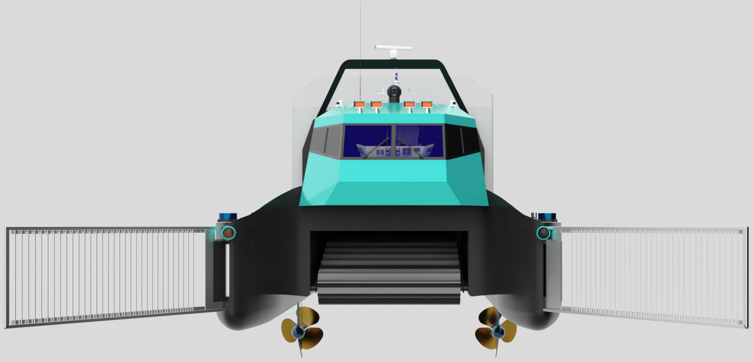

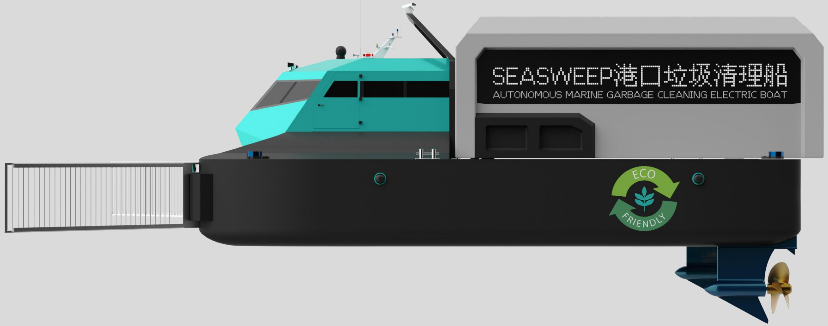

Dimensional data

LOA	13.5M
LWL	11.7 M
BEAM	3.3 M
Draft	1.3 M
Weight	3.0T
Load	1.5T

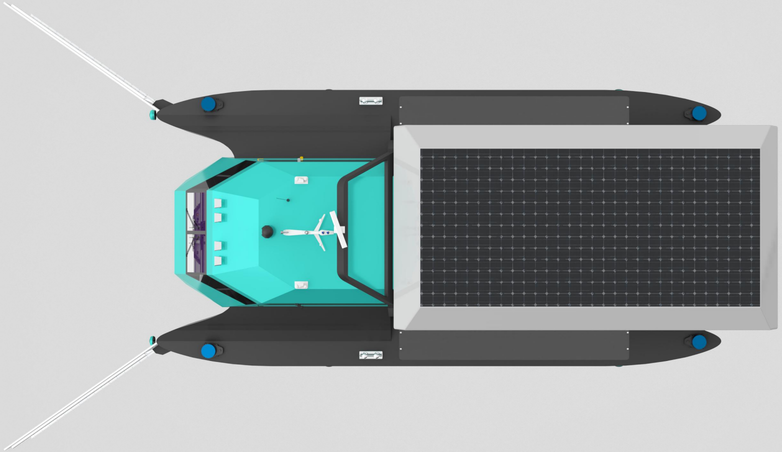

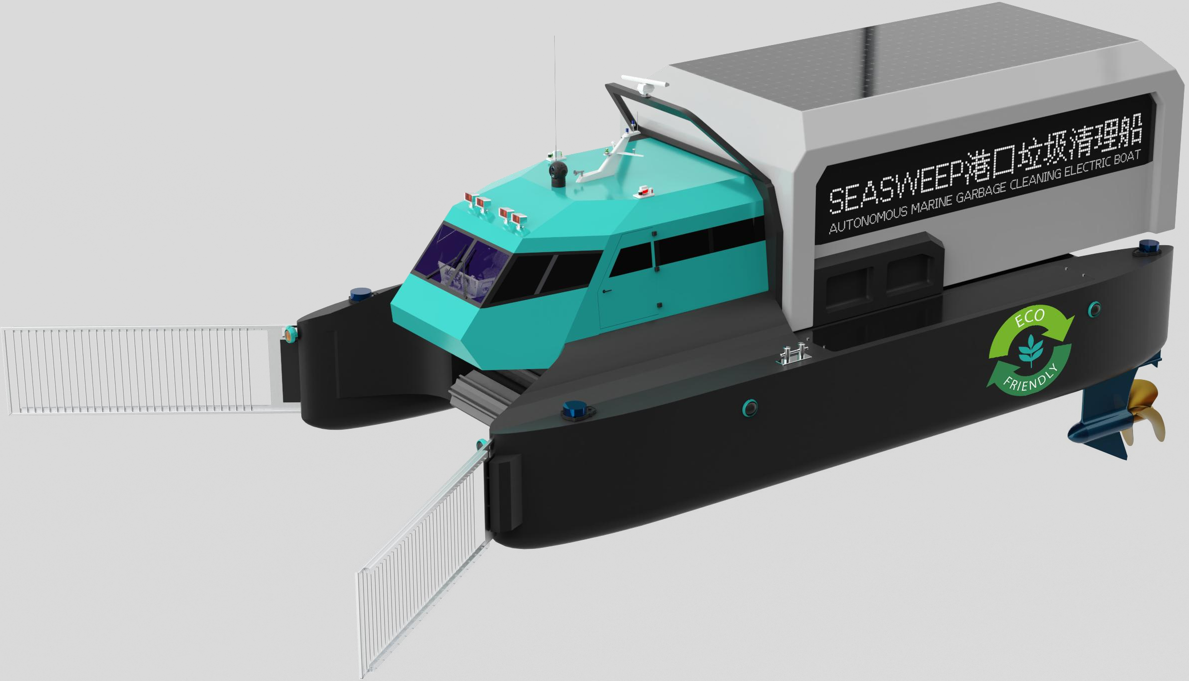
SEASWEEP港口垃圾清理船
AUTONOMOUS MARINE GARBAGE CLEANING ELECTRIC BOAT
ECO
FRIENDLY

Structure map

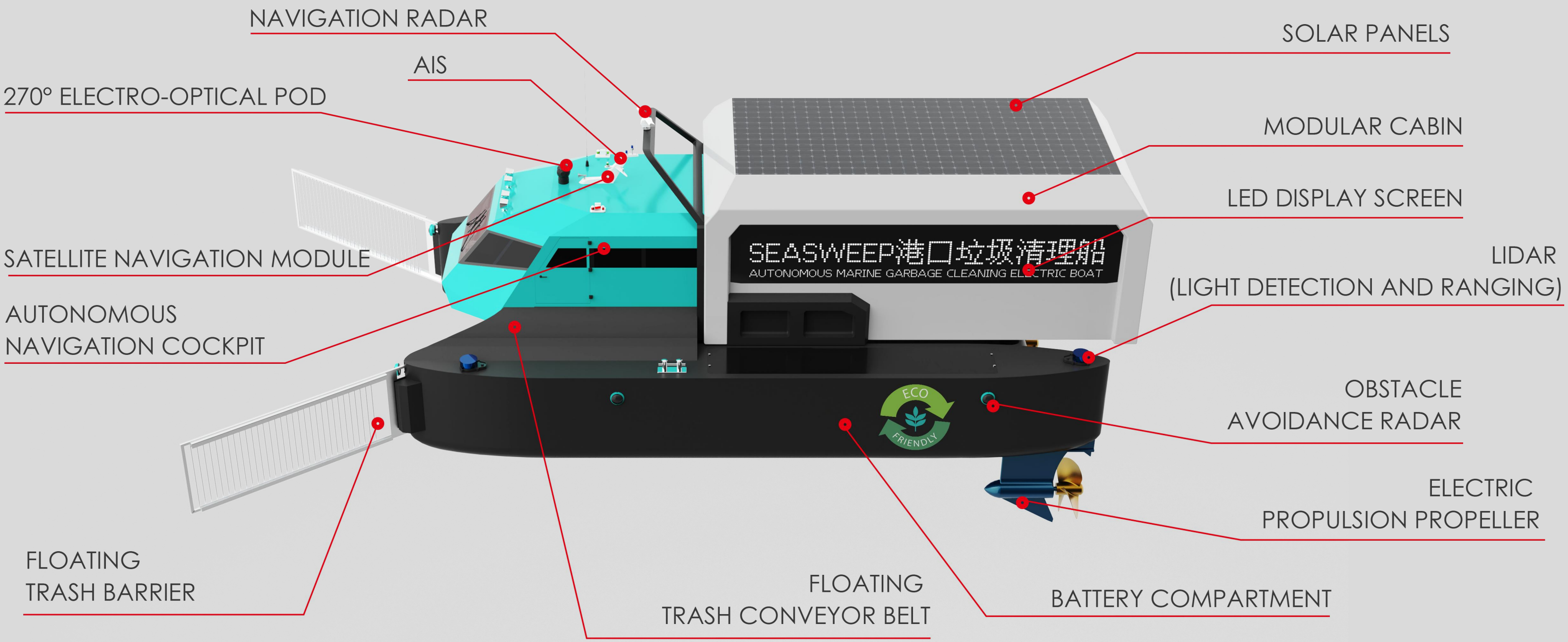

Function introduction

Autonomous Navigation

- The garbage cleaning ship can switch between autonomous navigation and manned operation. It operates at an autonomy level between L4 and L5. Utilizing environmental perception sensors and a cloud control system, the garbage cleaning ship can autonomously plan routes for cleaning within designated areas.

Sustainable Energy Management

- The cleaning ship is powered by Electric Propulsion Propeller and equipped with an optimized battery management system. Shore-based docks are fitted with energy storage and charging stations. Within the ship's overall energy system, 55% of the energy comes from the solar energy storage at the charging stations and the ship's own solar panels, achieving zero emissions and zero pollution.

Sustainable Waste Management System

- Surface floating debris is primarily composed of man-made waste and aquatic plants. Using an internal conveyor belt and sorting device, the ship initially classifies man-made debris (such as plastic bottles, fishing nets, and buoys) and biological waste (such as water weeds, algae, and animal carcasses). The collected waste is compressed on the ship and then transported to an onshore waste processing station for secondary sorting. Man-made waste with recycling value is processed and recycled, while biological waste, mostly aquatic plants, can be converted into fertilizer or livestock feed pellets. This process highlights the ship's commitment to sustainable waste management by efficiently recycling and repurposing collected materials.

ECO

Function introduction

Autonomous Navigation

- The garbage cleaning ship can switch between autonomous navigation and manned operation. It operates at an autonomy level between L4 and L5. Utilizing environmental perception sensors and a cloud control system, the garbage cleaning ship can autonomously plan routes for cleaning within designated areas.

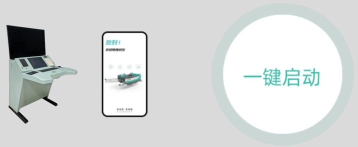

STEP 1

- Remote one-click/scheduled activation via mobile/PC

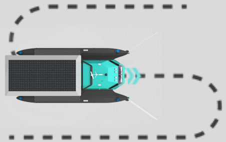

STEP 2

- Autonomous path tracking and cleaning operations

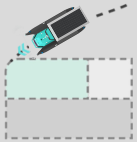

STEP 3

- Initiates unmanned area cleaning management

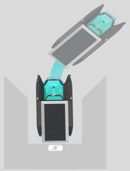

STEP 4

- Automatic return for recharging when fully loaded/low battery

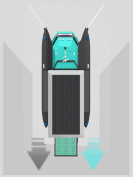

STEP 5

- Autonomous lifting of shore-side garbage bins

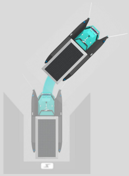

STEP 6

- Resumes automatic work mode after charging is complete

Function introduction

Autonomous Navigation

- The garbage cleaning ship can switch between autonomous navigation and manned operation. It operates at an autonomy level between L4 and L5. Utilizing environmental perception sensors and a cloud control system, the garbage cleaning ship can autonomously plan routes for cleaning within designated areas.

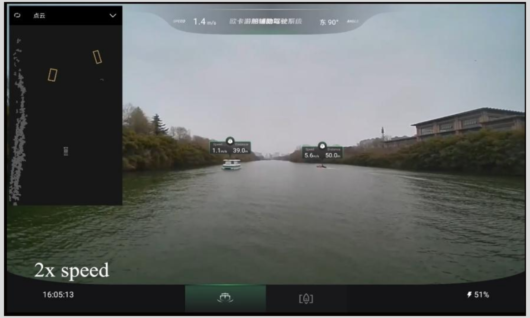

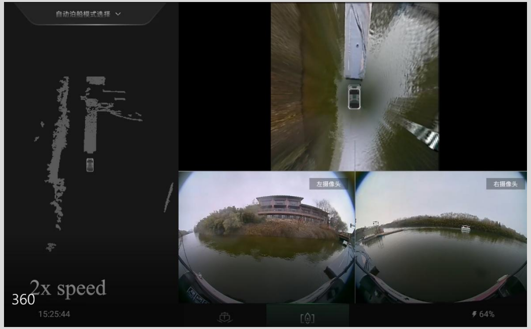

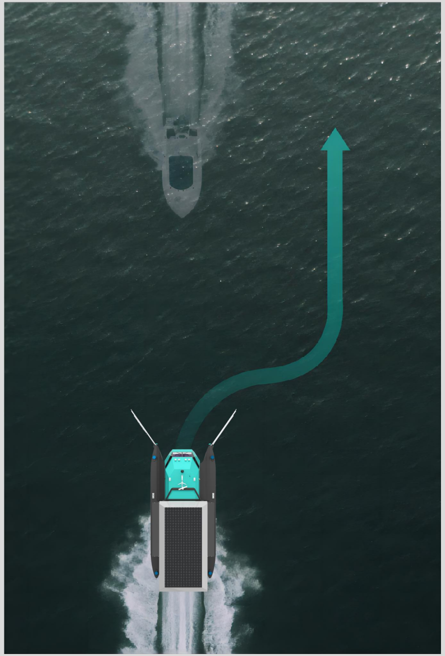

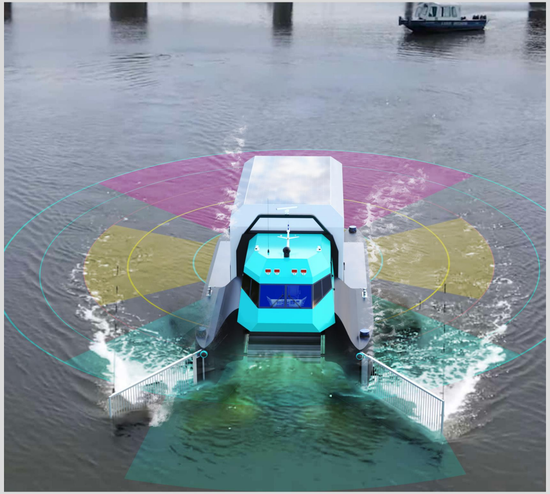

Function introduction

Sustainable Energy Management

- The cleaning ship is powered by Electric Propulsion Propeller and equipped with an optimized battery management system. Shore-based docks are fitted with energy storage and charg ing stations. Within the ship's overall energy system, 55% of the energy comes from the solar energy storage at the charging stations and the ship's own solar panels, achieving zero emissions and zero pollution.

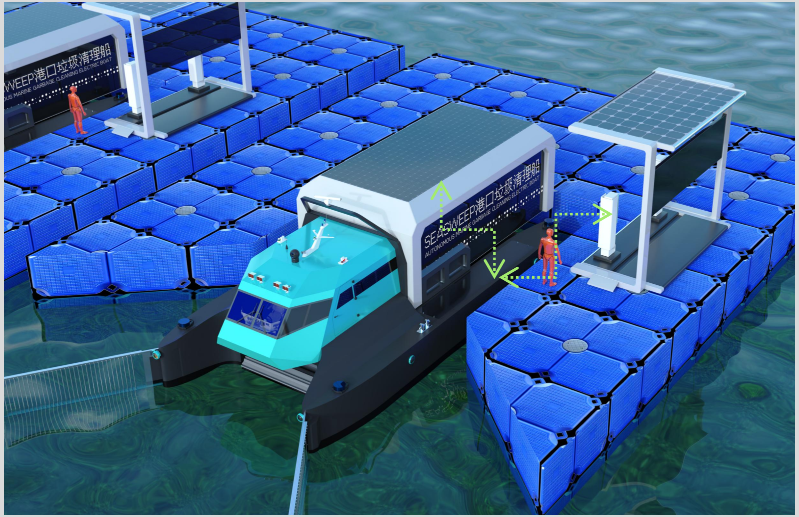

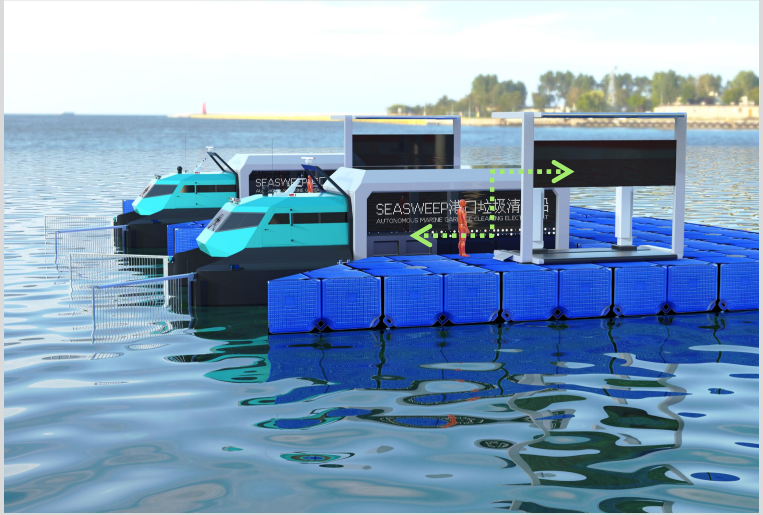

Function introduction

Sustainable Energy Management

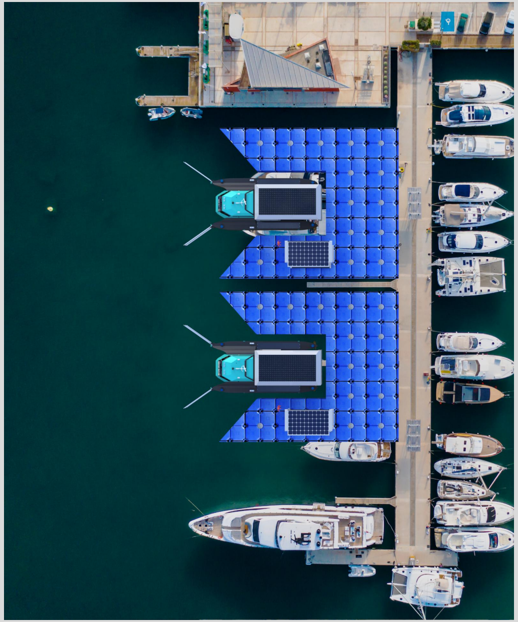

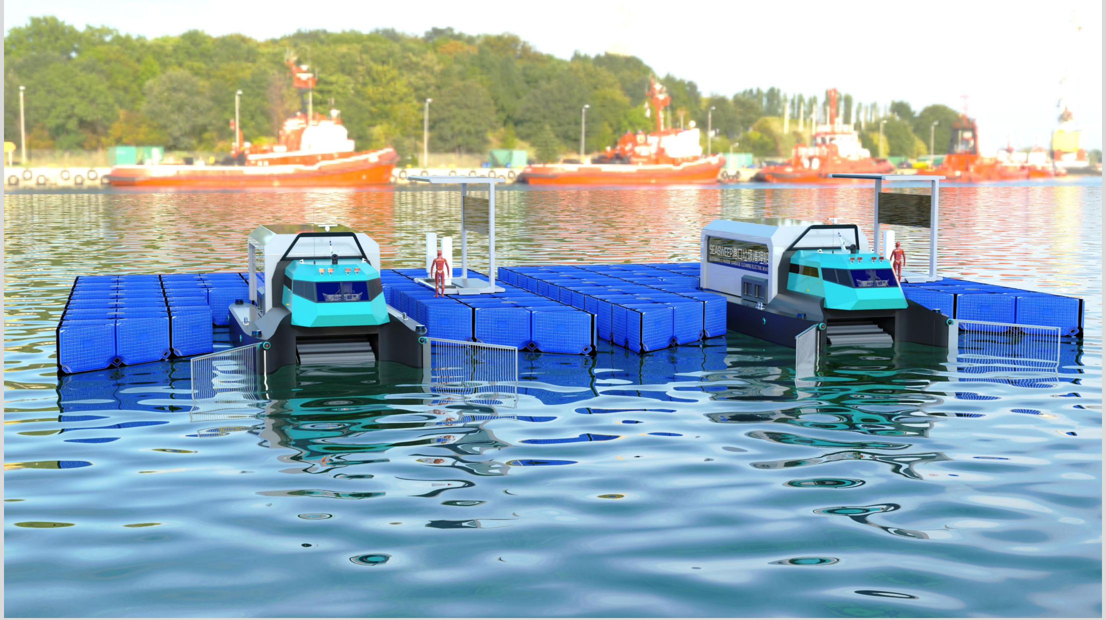

Function introduction

Sustainable Waste Management System

- Surface floating debris is primarily composed of man-made waste and aquatic plants. Using an internal conveyor belt and sorting device, the ship initially classifies man-made debris (such as plastic bottles, fishing nets, and buoys) and biological waste (such as water weeds, algae, and animal carcasses). The collected waste is compressed on the ship and then transported to an onshore waste processing station for secondary sorting. Man-made waste with recycling value is processed and recycled, while biological waste, mostly aquatic plants, can be converted into fertilizer or livestock feed pellets. This process highlights the ship's commitment to sustainable waste management by efficiently recycling and repurposing collected materials.

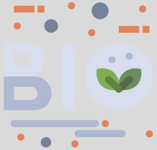

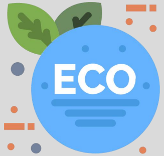

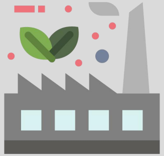

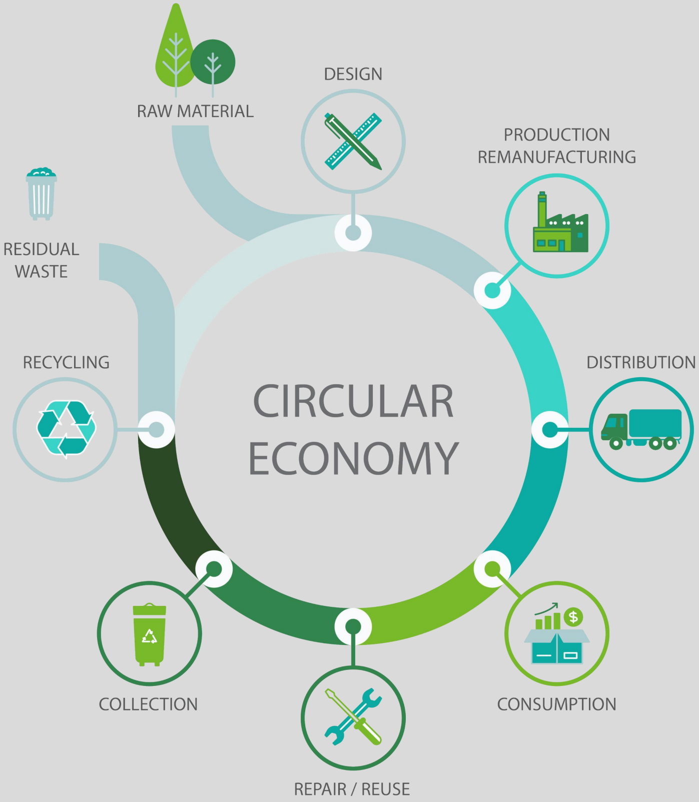

Function introduction

Sustainable Waste Management System

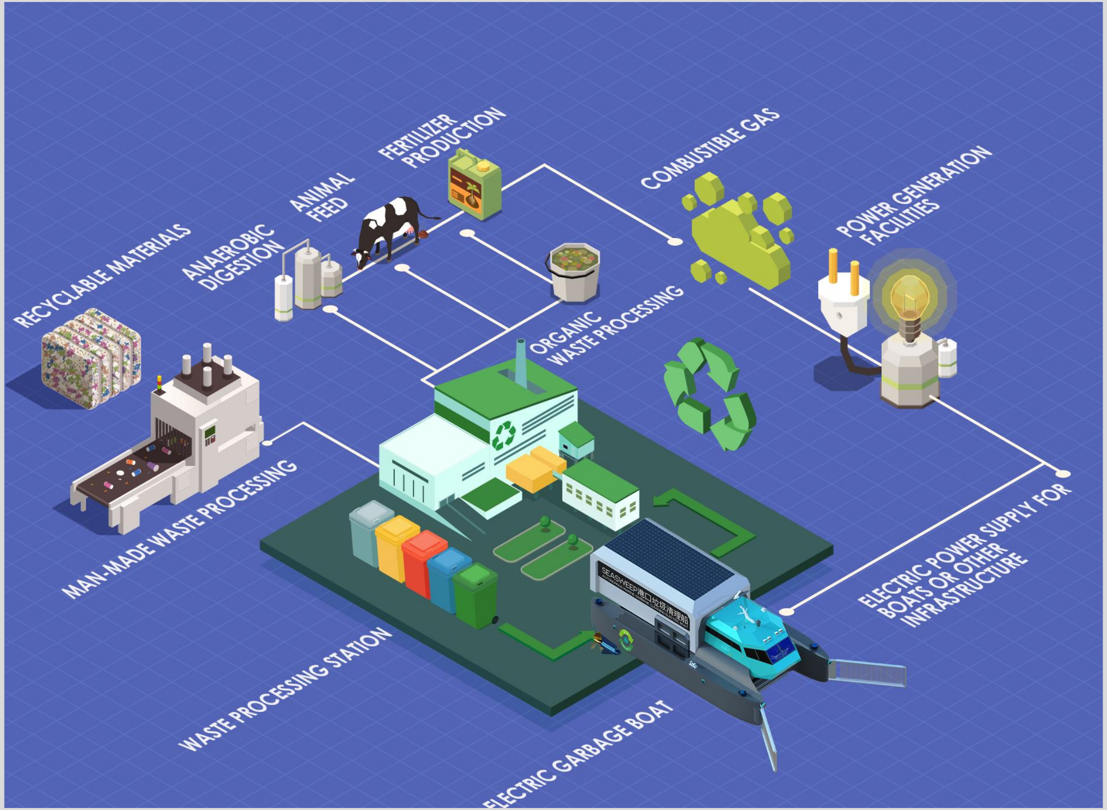

Usage scenario

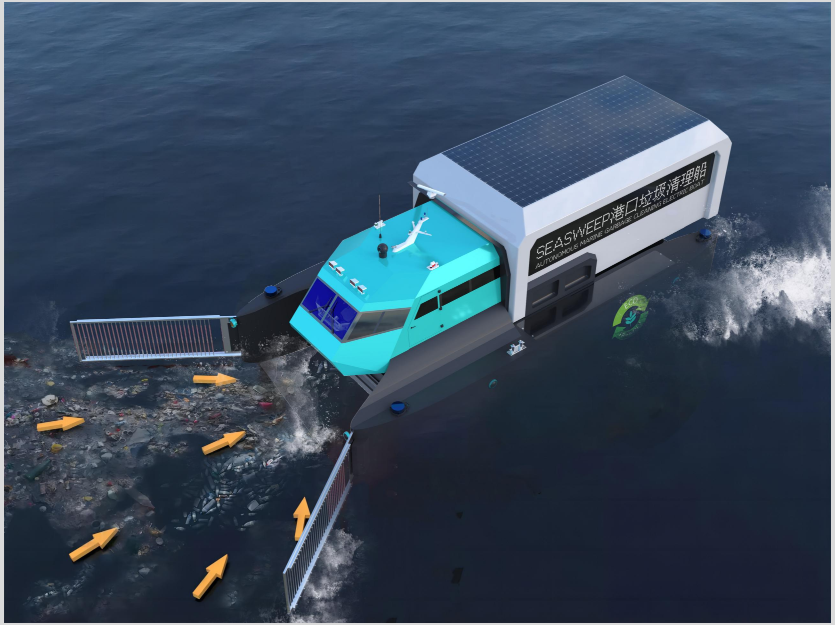

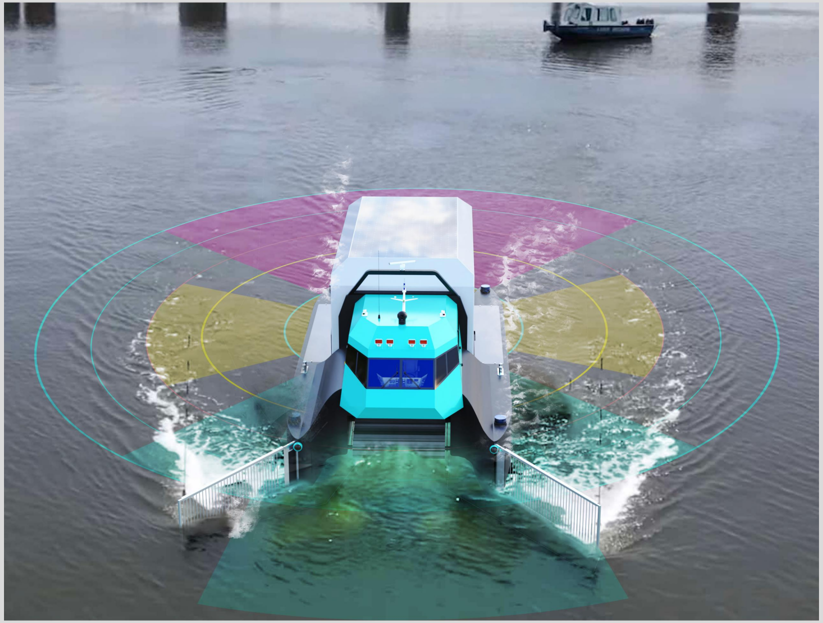

Usage scenario

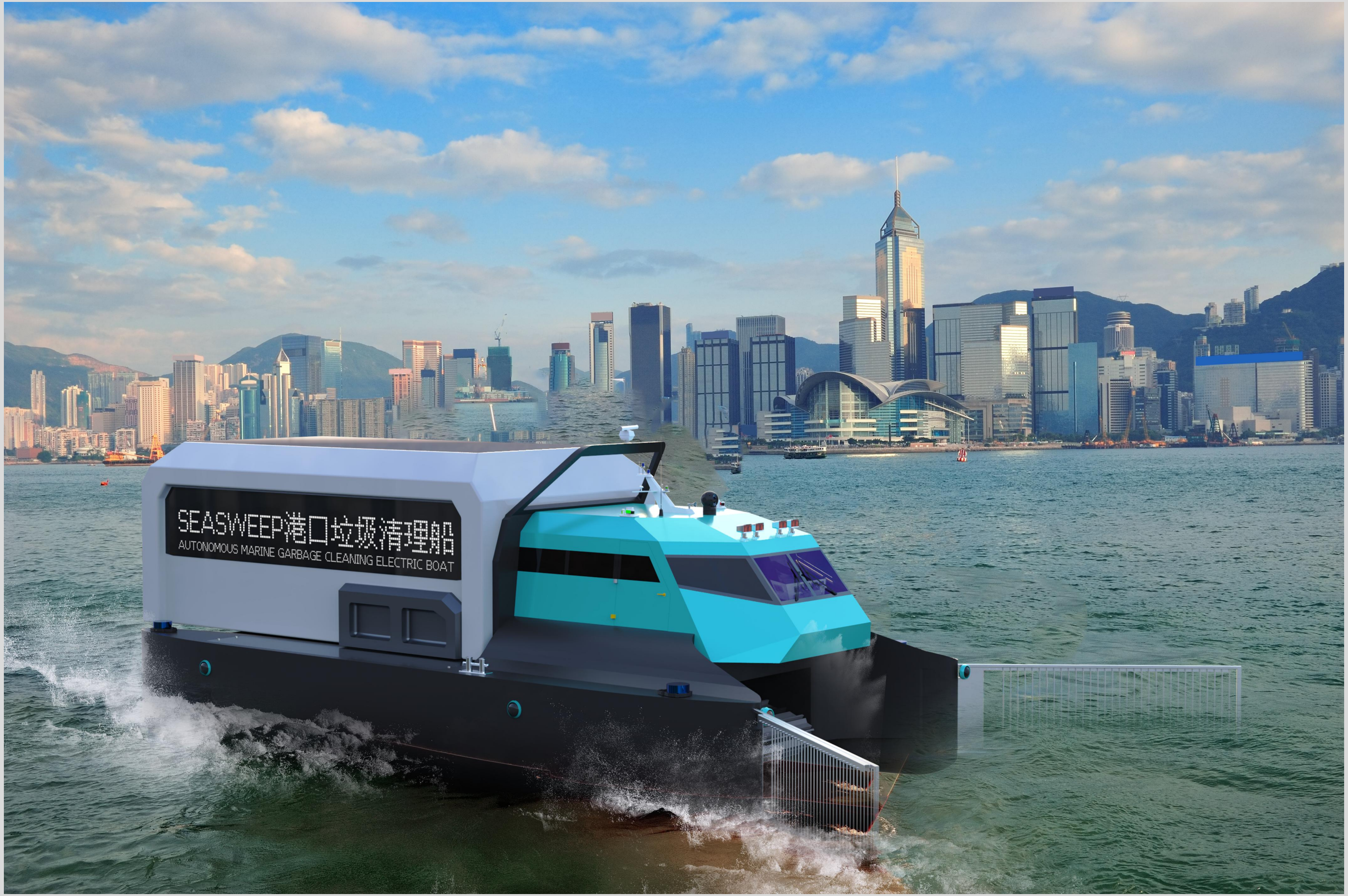

Usage scenario

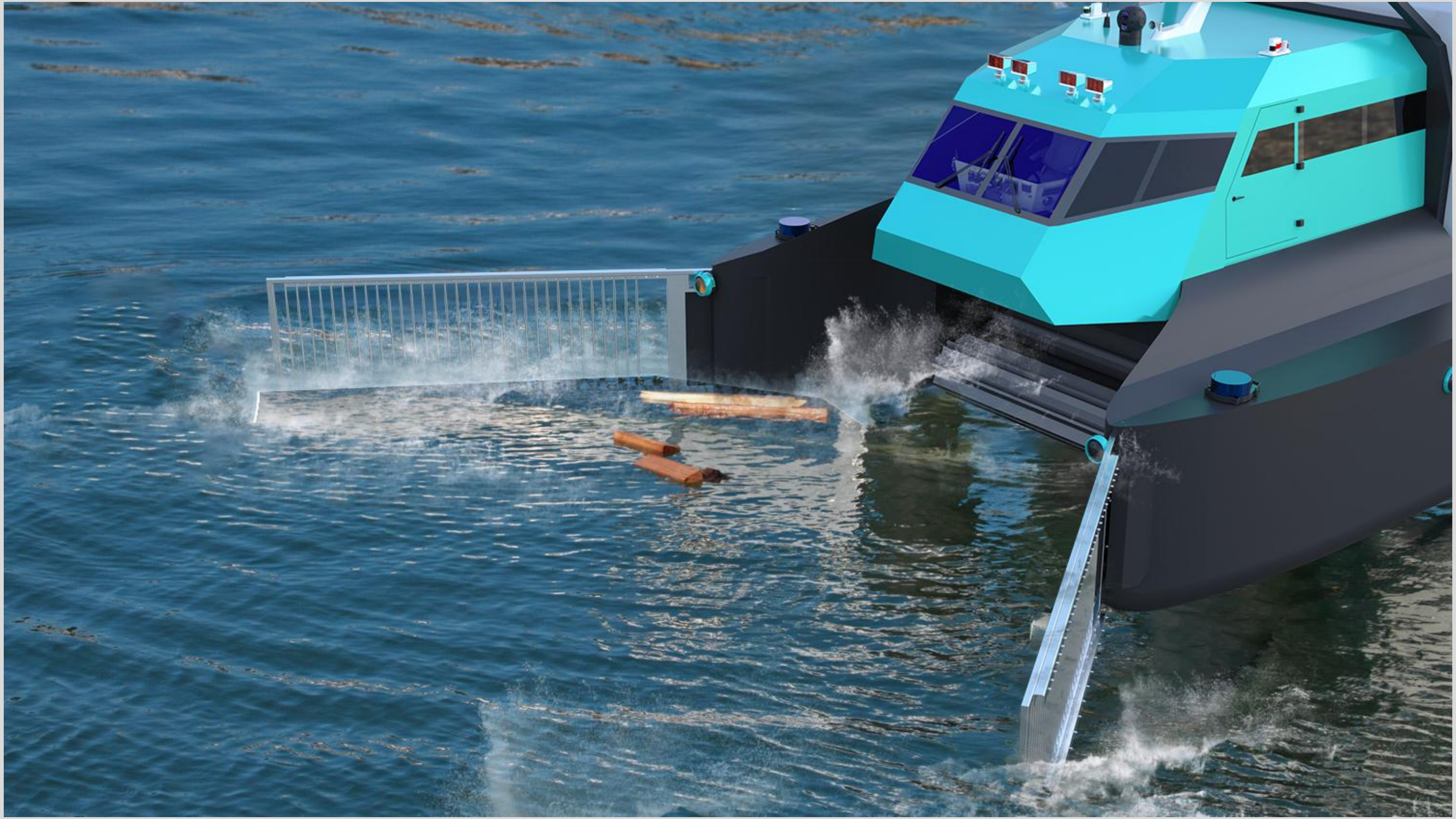

Usage scenario

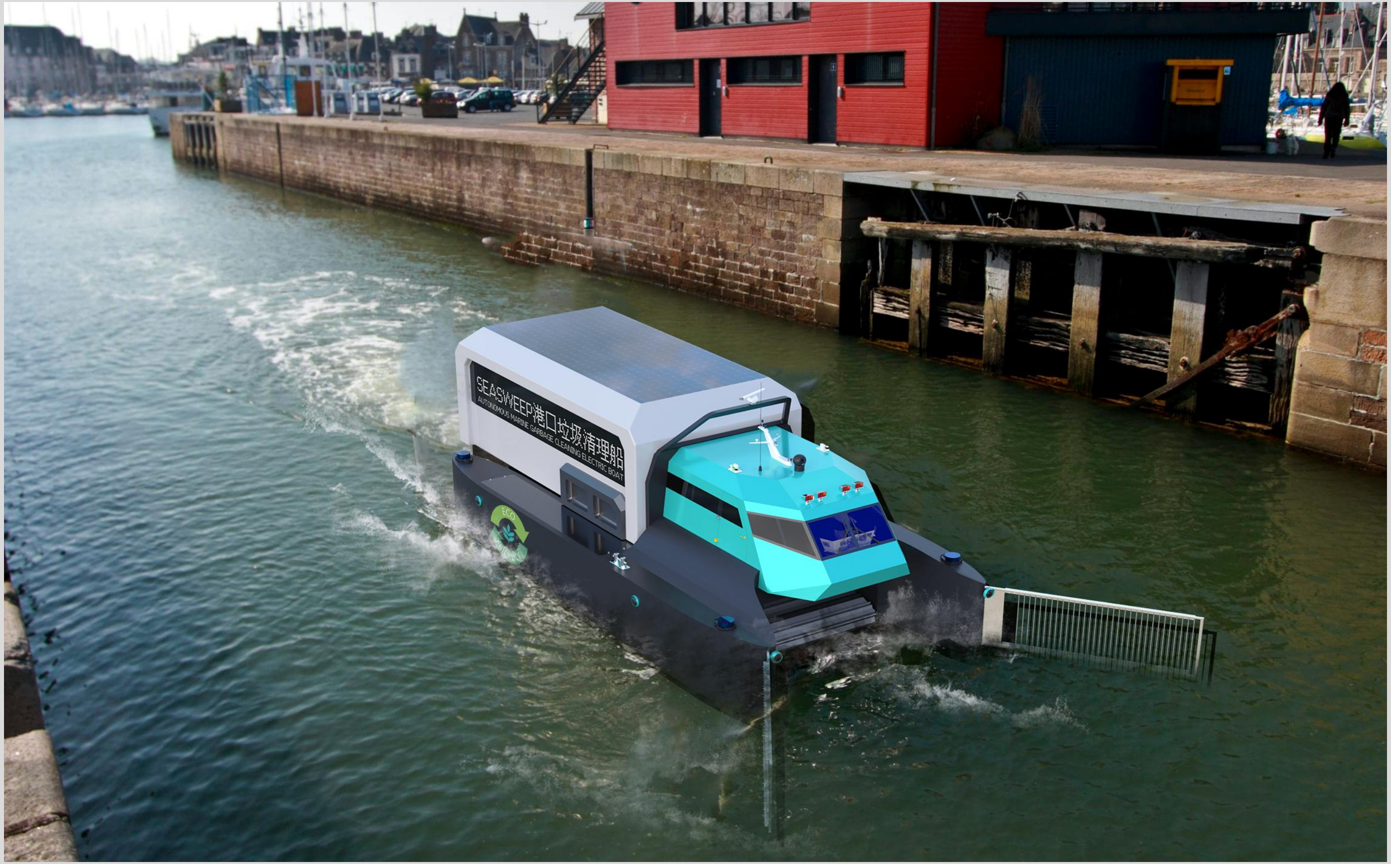

Supplement: Supplementary file 1 [file jemr-17-04-f-SD1-01.pdf]
